# Abstract of Report of Committee on Appliances and Improvements

**Published:** 1898-01

**Authors:** 


### Abstract of Report of Committee on Appliances and Improvements.

Read before Southern Dental Association, Old Point Comfort, August, 1897.

C. F. C. Mehlig's saliva controller offers the following advantages:

When placed in the mouth it keeps the tongue and cheek from the teeth, and forms a free space in which absorbents—as cotton rolls, wood pulp, spunk, bibulous paper, etc., may be placed. Both hands of the operator are left free; the rubber dam can be dispensed with; nerve canals in the lower teeth can be filled with no risk of saliva entering; the teeth are kept dry while crowns and small pieces of bridge-work are cemented to place. The patient can close the mouth to relieve the strain on the muscles and the teeth still be kept dry. Last, but not least, it is very durable; will last a life-time.

Dr. Parramore offered a new set of scalers, having the blade crescent-shaped, causing it to fit closely to the root and preventing wounding the tissues. He also presented a sterilizer devised by himself, a modification of the Moffit generator, depending upon formaldehyd gas generated by the decomposition of wood-alcohol.

Dr. W. E. Walker presented his facial clinometer.

Mr. N. J. Pohelman presented a means of applying a 24-k. gold surface to regulating appliances, etc., without the use of battery. It is instantaneous in action.

Dr. R. E. Payne presented a simple appliance for treating the antrum, consisting of a slender nozzle, three feet of rubber tubing, and a funnel or cup. The cup is filled below the level of the face, then raised gently, flushing the antrum, but avoiding the force accompanying the use of the syringe. Lowering the tube syphons out the solution acid.

Dr. H. S. Lowry presented a crown and bridge-work system—A series of dies, a neat, metallic stamper, a series of "trial caps," and a unique soldering plyer, constructed in such a way as to prevent tilting of the cap while allowing the heat to be distributed at all points of the crown alike.

Dr. J. M. Straut presented a universal matrix-holder; a convenient little instrument.

Dr. B. Holly Smith presented an ingenious gold-annealer.

Dr. Thos. Doltner presented a dental poultice, composed of hops, slippery elm, gum tragacanth, cayenne pepper, etc., put up in thin muslin bags, of size and shape convenient for application to the gum over the root of an affected tooth.

Dr. L. G. Noel introduced sterilized thorns of the prickly pear, as admirably adapted for filling root canals in connection with chloropercha. The thorns possess a springy flexibility most desirable, and grow in various sizes.

Dr. Gordon White, the Chairman of the Committee, presented a set of four very thin burnishers of different forms; also, pair of pliers for the application of medicaments to gum or cavities. They are "cross-action" pliers, formed from the Nos. 13 and 17, White's catalogue, designed to hold the medicated cotton while the gum or cavity is being dried out, breaking up the habit of putting foil pliers between the teeth to hold the cotton while preparation or its application is being made.

